# Neuroimaging of Meckel’s cave in normal and disease conditions

**DOI:** 10.1007/s13244-018-0604-7

**Published:** 2018-04-18

**Authors:** Ajay Malhotra, Long Tu, Vivek B. Kalra, Xiao Wu, Ali Mian, Rajiv Mangla, Elias Michaelides, Pina Sanelli, Dheeraj Gandhi

**Affiliations:** 1 Department of Radiology and Biomedical Imaging, Yale School of Medicine, Box 208042, Tompkins East 2, 333 Cedar St, New Haven, CT 06520-8042 USA; 2 Orlando Health, Orlando, FL USA; 3 State University of New York Upstate Medical University, Syracuse, NY USA; 4 Department of Surgery (Otolaryngology) and Department of Pediatrics, Yale School of Medicine, New Haven, CT USA; 5 Northwell Health, Great Neck, NY USA; 6 Department of Radiology, University of Maryland Medical Center, Baltimore, MD USA

**Keywords:** Meckel’s cave, Trigeminal, Neuralgia, Perineural, Skull base

## Abstract

**Abstract:**

Meckel’s cave is a dural recess in the posteromedial portion of the middle cranial fossa that acts as a conduit for the trigeminal nerve between the prepontine cistern and the cavernous sinus, and houses the Gasserian ganglion and proximal rootlets of the trigeminal nerve. It serves as a major pathway in perineural spread of pathologies such as head and neck neoplasms, automatically upstaging tumours, and is a key structure to assess in cases of trigeminal neuralgia. The purpose of this pictorial review is threefold: (1) to review the normal anatomy of Meckel’s cave; (2) to describe imaging findings that identify disease involving Meckel’s cave; (3) to present case examples of trigeminal and non-trigeminal processes affecting Meckel’s cave.

**Teaching points:**

• *Meckel’s cave contains the trigeminal nerve between prepontine cistern and cavernous sinus.*

• *Assessment is essential for perineural spread of disease and trigeminal neuralgia.*

• *Key imaging: neural enhancement, enlargement, perineural fat/CSF effacement, skull base foraminal changes.*

## Introduction

Meckel’s cave is a natural mouth-shaped aperture in the medial portion of the middle cranial fossa that acts as a key conduit for the largest cranial nerve, the trigeminal nerve (CN V). It connects the cavernous sinus to the prepontine cistern of the posterior fossa. This tiny parasellar T2-hyperintense cerebrospinal fluid (CSF)-containing structure holds tremendous importance in neuroimaging—its effacement or abnormal enhancement may herald otherwise occult infectious, inflammatory, congenital or neoplastic lesions. Its careful assessment can lead to early detection of perineural spread of malignancy with significant prognostic and therapeutic implications.

## Anatomy

**Fig. 1:**
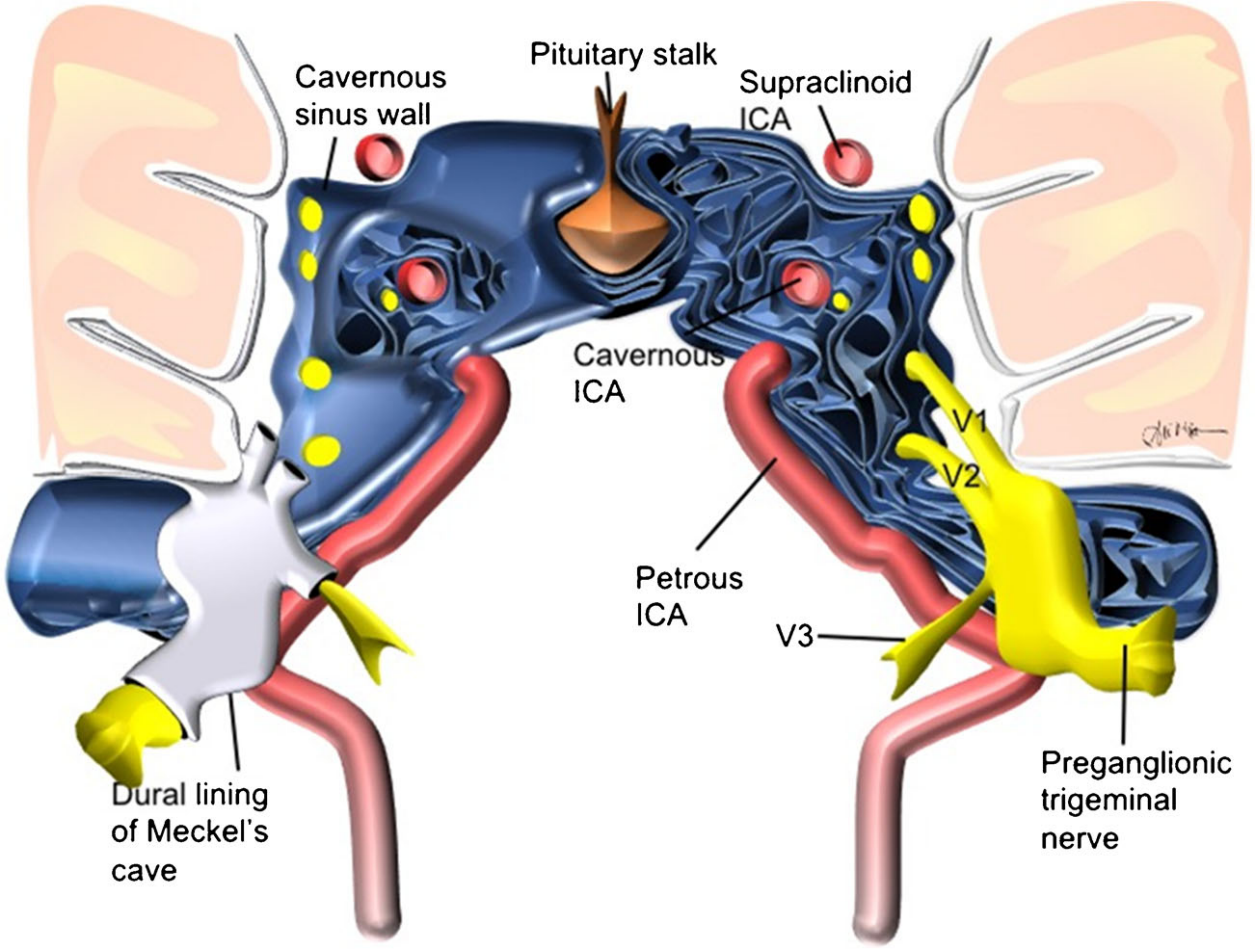
Schematic representation of Meckel’s cave and relationship with adjacent structures

Meckel’s cave is an aperture within petrous apex’s meningeal dura propria and periosteal layers measuring 4 × 9 mm wide at its opening and 15 mm in length [10]. The cave is shaped like an open-ended three-fingered glove pointing anterosuperomedially (Fig. 1). The palm of the glove rests within a bony indentation of the petrous apex (impressio trigemini) and contains the semilunar-shaped Gasserian ganglion of the trigeminal nerve. The fingers of the glove, superior to inferior, contain the three postganglionic rootlets that comprise the “tri-” of the trigeminal nerve—ophthalmic (V1), maxillary (V2) and mandibular (V3), which provides sensory innervation to the face and motor function for mastication. The cuff of the glove or the entrance of the cave, the porus trigeminus, is between the superior and inferior petrosal sinuses and contains the trigeminal nerve with an arachnoid sheath [21]. The internal carotid artery precavernous segment courses inferomedial to the cave. Anteriorly, lies the cavernous sinus, with its lateral wall superomedial to the cave [18]. Trigeminal nerve (CN 5) branches V1 and V2, oculomotor nerve (CN 3) and trochlear nerve (CN 4) course within a dural sheath that comprises cavernous sinus lateral wall itself. V1 exits through the superior orbital fissure with the oculomotor, trochlear and the abducens nerves (CN 6), and receives sensory input from the eye, orbit and forehead. V2 exits through foramen rotundum, an imaging landmark in the sphenoid bone superolateral to the vidian canal, and receives sensory input from the maxilla, palate, upper lip, cheek, nasal cavity, nose and nasopharynx. V3 exits inferiorly between Meckel’s cave and the cavernous sinus through the foramen ovale, coursing down towards the mandible, and receives sensory input from the chin, lower lip, floor of mouth, tongue, scalp and meninges, and gives motor output to the masticator muscles (masseter, medial pterygoid, lateral pterygoid, temporalis), tensor veli palatini and tensor tympani. Perineural vascular plexus surrounds the Gasserian ganglion and proximal V2 and V3 rootlets, resulting in normal findings of thin mild enhancement [23].

## Imaging modalities and imaging technique

Magnetic resonance imaging (MRI) is preferred to assess Meckel’s cave, its contents and relationship with adjacent structures. Dedicated, high-resolution images from the orbital apex through the prepontine cistern are obtained. Parallel imaging and high-field 3-T MRI depict fine anatomical detail, especially the cranial nerves and walls of the Meckel’s cave. The MRI protocol should include imaging in three planes with T1- and T2-weighting, short-tau inversion recovery (STIR) and gadolinium-enhanced T1-images with fat suppression. STIR, which is not based on frequency-selective pulses, is preferred for more homogeneous fat suppression at the skull base [4]. High-resolution heavily T2-weighted volumetric sequences can demonstrate the cisternal course of the cranial nerves as well as the trigeminal rootlets and ganglion within Meckel’s cave [5]. A thin-section (3 mm), small field of view (FOV) (16–18 cm), fast spin echo, T1-weighted sequence is best to depict bone marrow invasion and assess fat planes at the skull base. Intravenous gadolinium-enhanced fat-suppressed T1-weighted high-resolution, small FOV images depict meningeal invasion and perineural spread and maximise tumour contrast against adjacent structures.

Computed tomography (CT) better defines the bony anatomy of the skull base and the thin cortical margin of the skull base foramina, thereby providing information about aggressiveness of a lesion. Multi-detector helical CT acquisition in the axial plane with reformats in the sagittal and coronal planes are routinely displayed as 3-mm slices, with thinner sub-millimetre slices available as needed.

## Imaging indications and key features

Skull base imaging specifically evaluating Meckel’s cave is most frequently performed for assessing for trigeminal perineural spread of head and neck malignancy and trigeminal neuralgia. Trigeminal neuropathy may present as facial pain, numbness and weakness of muscles of mastication, and even trismus. Meckel’s cave may be involved in a spectrum of pathologies: congenital, infectious, inflammatory, vascular or neoplastic lesions (Tables 1 and 2). Key imaging features of pathology of Meckel’s cave are moderate enhancement greater than the perineural vascular plexus, nerve enlargement with perineural fat plane effacement, osseous foraminal erosion or enlargement, and trigeminal cistern CSF effacement. Any enhancement of the trigeminal nerve posteriorly, within the cisternal segment or root entry zone, is a specific sign of pathological enhancement. Foraminal assessment holds a greater role in assessing for retrograde perineural spread, which can occur discontinuously, with cisternal/root entry zone enhancement portending a worse prognosis [15].

**Table 1:** Trigeminal and non-trigeminal entities involving Meckel’s cave

Trigeminal	Common - Perineural spread of cancers	Uncommon - Nerve sheath tumours, CIDP - Infection - Herpes
Non-trigeminal	Common - Meningioma - Leptomeningeal metastasis	Uncommon - Vascular - Persistent trigeminal artery, vascular loops - Lymphoma - Sarcoid - Pituitary macroadenoma - Petrous mucocele/cephalocele - Cholesterol granuloma/cholesteatoma/epidermoid - Chondroid lesions/chordoma - ICA aneurysms - Tolosa-Hunt

**Table 2:** Entities involving Meckel’s cave

Developmental	- Arachnoid cyst - Meckel’s cave encephalocele	
Vascular	- Persistent trigeminal artery - Vascular loop - trigeminal AVM	
Inflammatory	- Sarcoidosis - Herpes simplex virus - Tuberculosis	
Neoplastic	Benign	- Schwannomas - Meningiomas
	Malignant	- CSF spread of primary CNS malignancies - Lymphoma - Perineural spread
Miscellaneous	Non-intrinsic lesions of Meckel’s cave that may compress of invade Meckel’s cave	- Petrous apex mucocele - Pituitary macroadenoma

## Trigeminal disease

### Perineural spread

**Fig. 2:**
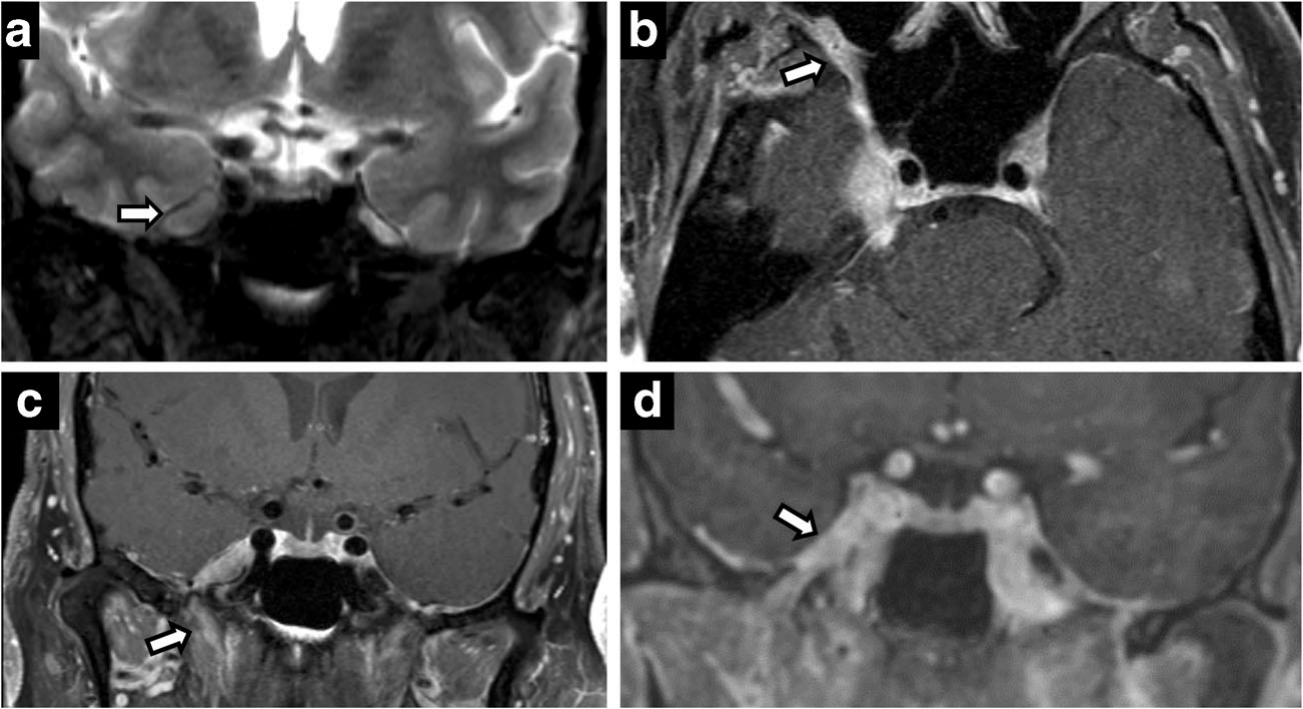
Perineural spread of tumour. Coronal T2 (**a**), axial C+ (**b**), coronal C+ (**c**), coronal C+ (**d**). A 60-year-old man with right CN V symptoms. Expansile T2 hypointense, enhancing lesion filling the right Meckel’s cave extending through the foramen ovale and along V2 in the foramen rotundum

**Fig. 3:**
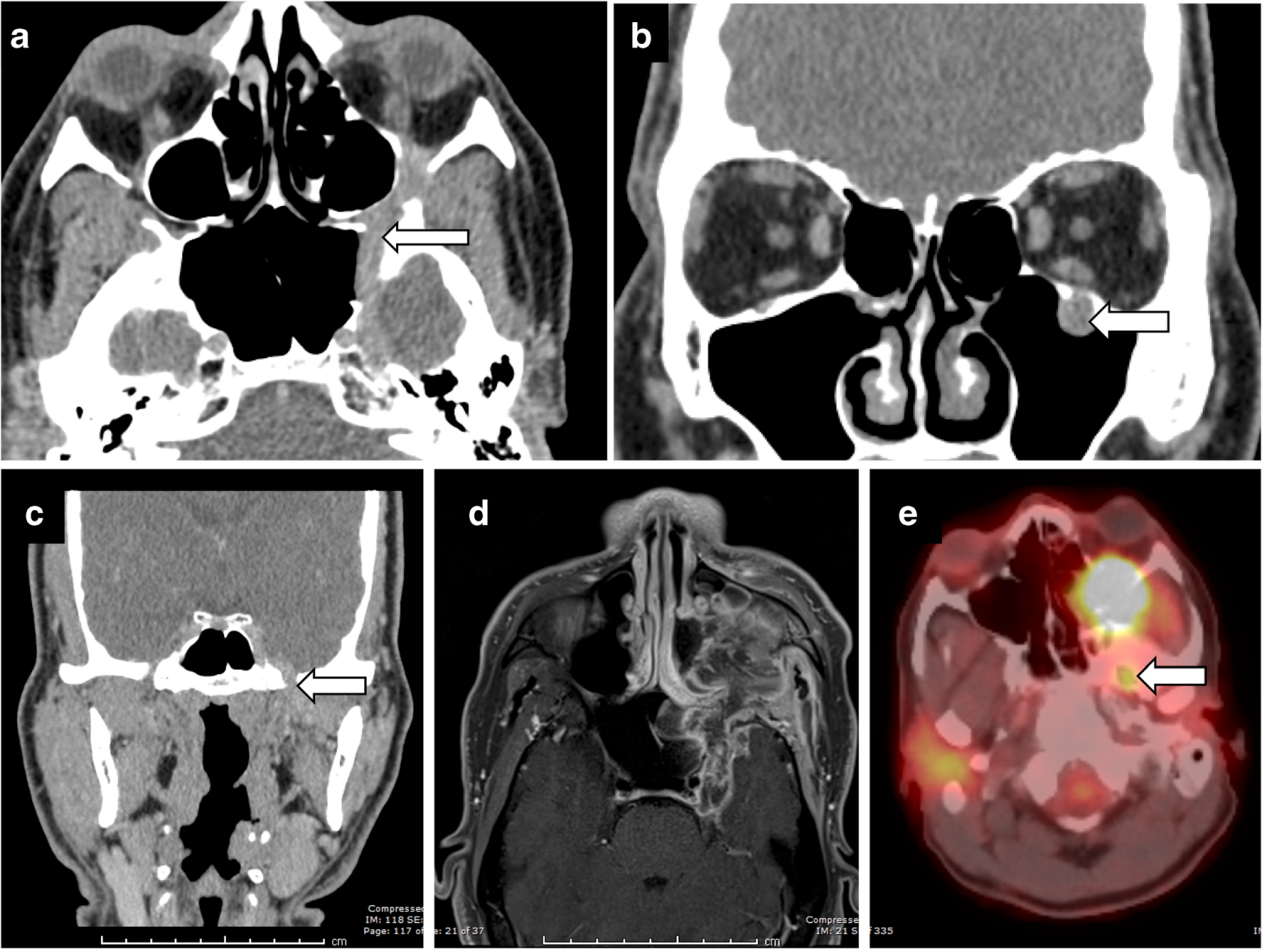
Perineural spread of tumour. A 45-year-old man with left facial pain and numbness. CT showing enhancing soft tissue along the course of left CN V, expanding into the foramen rotundum (**a**), infraorbital foramen (**b**) and foramen ovale (**c**). Posteriorly, the enhancing tumour extends to the left Meckel’s cave. Rapid expansion on follow-up MRI (**d**) with intense uptake on positron emission tomography (PET) (**e**) in left CN V including foramen ovale (*arrow*)

**Fig. 4:**
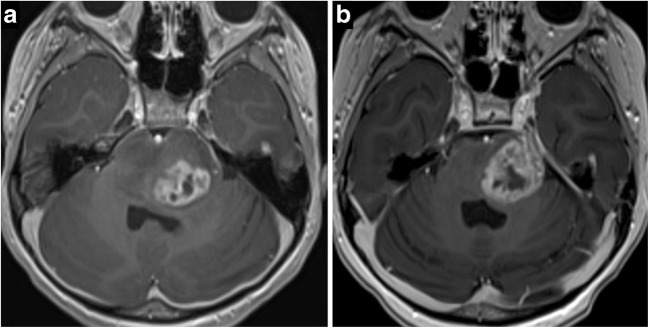
Diffuse infantile pontine glioma (**a**) with significant increase in size on the 3-month study (**b**) with perineural spread along left CN V into Meckel’s cave

Perineural spread from skin and head-and-neck malignancies can occur along the trigeminal and facial nerves. Its presence has marked implications for staging and treatment of otolaryngological malignancies, automatically upstaging tumours to T3 in the most recent eighth edition of American Joint Committee Cancer staging manual [12]. Maxillary nerve (V2) perineural spread occurs from primary tumours in the midface skin, maxilla, upper lip and palate. Mandibular nerve (V3) perineural spread occurs from tumours in the lower face, mandible, masticator space and parapharyngeal space. Trigeminal perineural involvement may also occur from spread along nerves communicating with facial nerve branches such as the greater superficial petrosal/vidian nerves near the pterygopalatine fossa and auriculotemporal nerve near the temporomandibular joint. Squamous cell carcinoma is the most common cause of perineural spread given its large prevalence, but adenoid cystic carcinomas of the minor salivary glands have the highest incidence [17] (Figs. 2 and 3). Brainstem tumours can also rarely spread anteriorly through CN V (Fig. 4).

### Trigeminal neuralgia

**Fig. 5:**
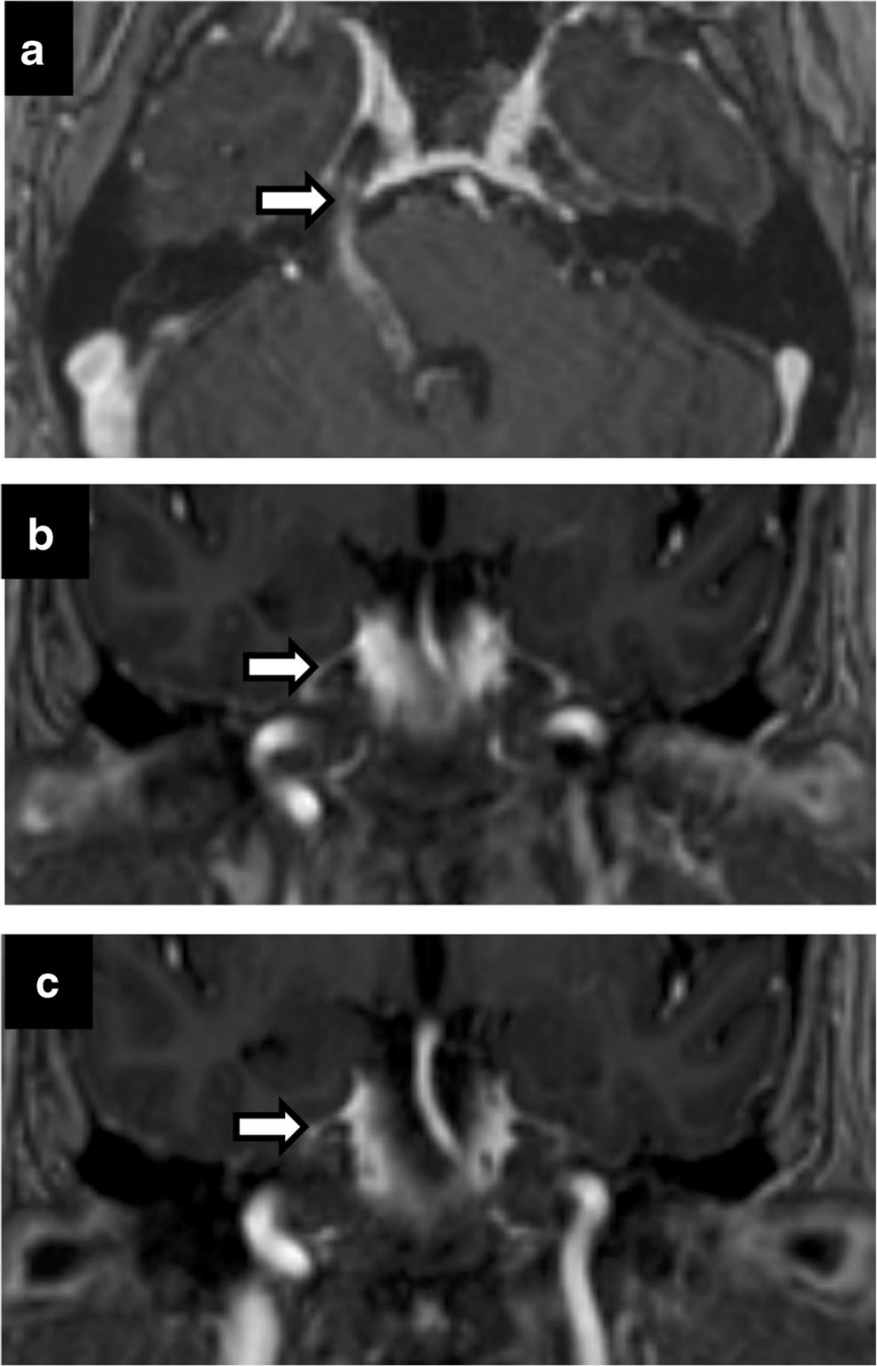
Herpes zoster reactivation. A 62-year-old woman with right perioral numbness for 2 weeks and vesicular rash. Postcontrast axial (**a**) and coronal (**b**, **c**) images showing tubular enhancement along the right CN V from the origin, through the cisternal segment and extending to Meckel’s cave

Although trigeminal neuralgia is a clinical diagnosis, neuroimaging may be performed for confirmation in viral/idiopathic aetiologies and to assess for treatable neurovascular compression. Viral aetiologies, including herpes zoster and simplex viruses, involve the Gasserian ganglion, where they can lie dormant. Mild enhancement of the ganglion is non-specific and difficult to distinguish from normal perineural vascular plexus; however, cisternal/root entry zone enhancement is specific. Herpes rhombencephalitis asymmetric enhancement in the clinical context of reactivation has characteristic imaging findings (Fig. 5).

**Fig. 6:**
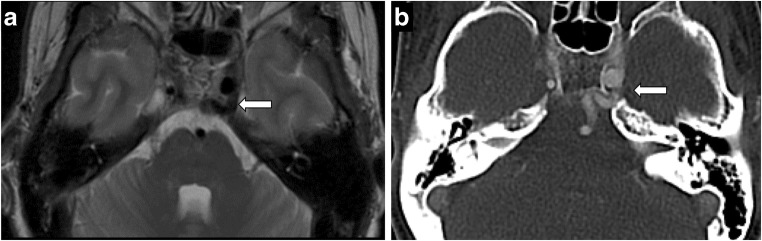
Persistent trigeminal artery. Axial T2-weighted imaging (**a**) and CT angiography (**b**). Vascular channel connecting the left internal carotid artery and the basilar artery, and running through the medial aspect of Meckel’s cave

**Fig. 7:**
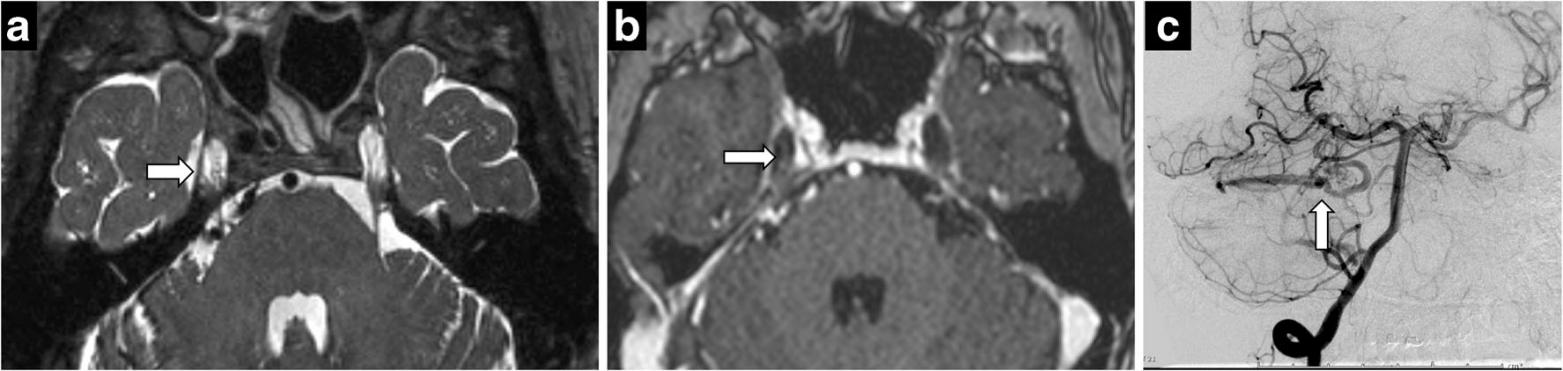
Trigeminal AVM. A 58-year-old man with right facial neuralgia. Axial T2 (**a**) and axial C+ (**b**) showing abnormal serpentine vasculature along cisternal segment of right CN V extending into Meckel’s cave on DSA (**c**)—arteriovenous shunting from right anterior inferior cerebellar artery (AICA) with prominent draining vein

Neurovascular compression can be suggested in the appropriate clinical context with trigeminal cisternal/root entry zone deformation from a vascular loop such as the superior cerebellar artery or anterior inferior cerebellar artery [9]. Persistent trigeminal artery, the most common persistent fetal carotid-basilar anastomosis, normally runs through a dural foramen located immediately medial to the Meckel’s cave (Fig. 6). Rarely, a vascular loop may be associated with trigeminal neural arteriovenous malformation, where symptoms may be from the malformation itself or nerve compression/deformation from enlarged feeding and draining vessels [11] (Fig. 7). Microvascular decompression is an effective treatment for these cases, although stereotactic radiosurgery has also been used, especially in the context of arteriovenous malformation (AVM). Non-vascular/idiopathic causes of trigeminal neuralgia are treated with anticonvulsants, antispasmodics and botox. Radiosurgery (gamma knife) is reserved for medically refractory symptoms. Enhancement without expansion can be transient or persistent following stereotactic radiosurgery [16].

### Neoplastic, inflammatory and other

**Fig. 8:**
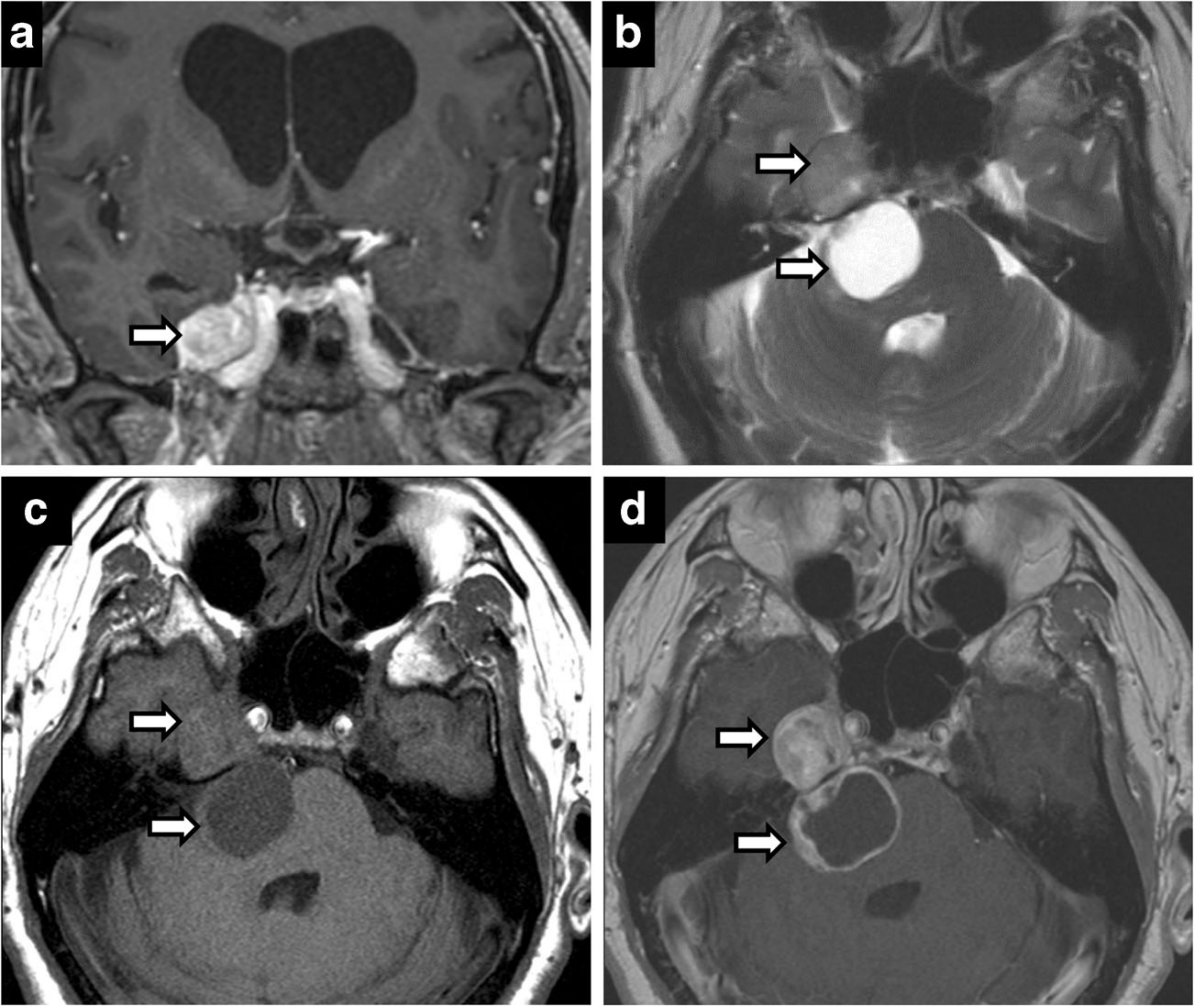
Trigeminal schwannoma. Coronal C+ (**a**), axial T2 (**b**), axial T1 (**c**), axial C+ (**d**): expansile enhancing mass in the right Meckel’s cave with a large, lobulated cystic component along the cisternal segment of the right CN V

**Fig. 9:**
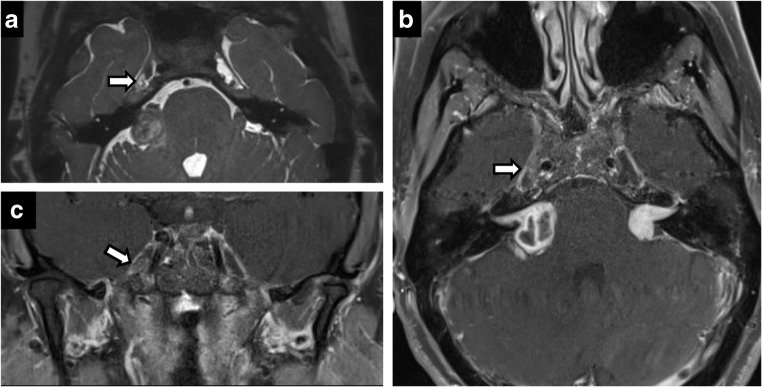
Schwannoma of Meckel’s cave in a patient with neurofibromatosis type 2. Axial T2 (**a**), axial C+ (**b**), coronal C+ (**c**) showing an enhancing lesion within the right Meckel’s cave. Note additional bilateral vestibular schwannomas

**Fig. 10:**
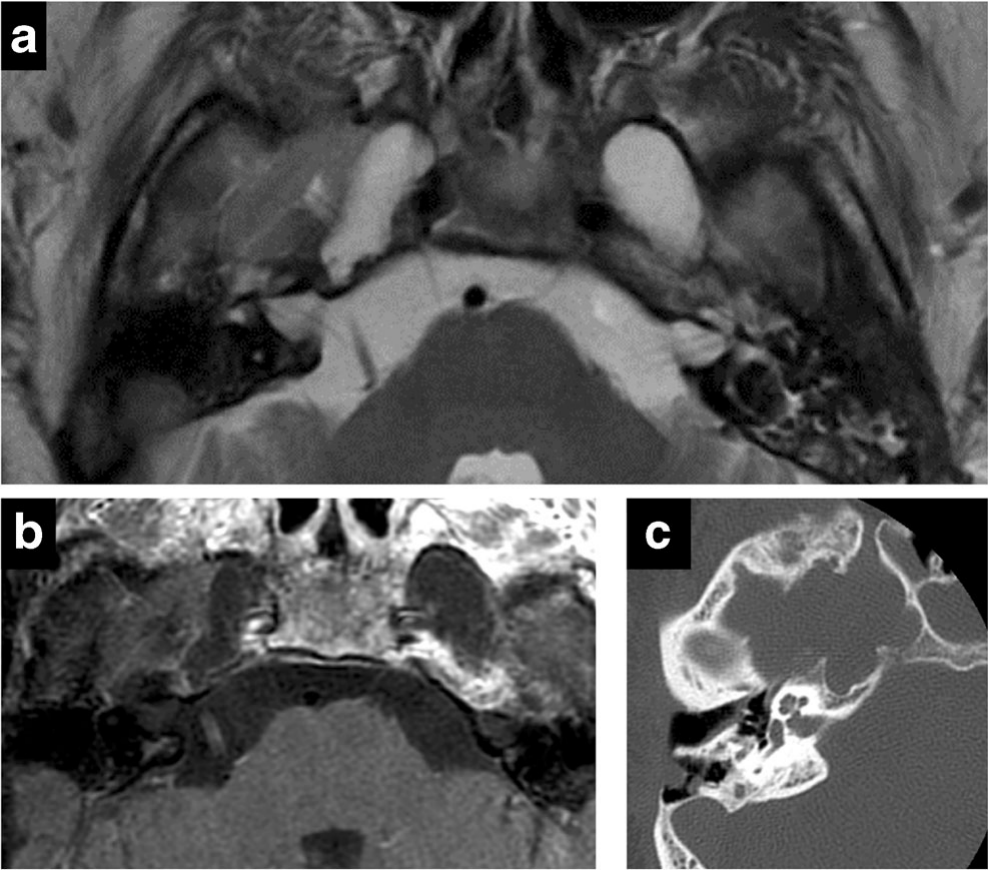
Dural ectasia in neurofibromatosis type 1. Axial T2 (**a**), axial C+ (**b**), CT (**c**). Bilateral enlargement of Meckel’s cave—CSF isointense and no abnormal enhancement. Smooth scalloping and remodelling of petrous apex on CT (**c**)

Predictably, the most common neoplasm of Meckel’s cave is a trigeminal nerve sheath tumour, schwannoma and neurofibroma. Nerve sheath tumours result in nerve and foraminal enlargement, demonstrate T2 hyperintense signal with moderate-to-intense heterogeneous enhancement. A dumbbell shape provides specificity, with the waist at constricting foramina (Fig. 8). In contrast, meningiomas often display T2 hypointense signal and show uniform, avid enhancement. Nerve sheath tumours may be isolated or syndromic, in phacomatoses such as neurofibromatosis (Fig. 9). Neurofibromatosis should be considered in cases of multiple nerve sheath tumours and dural ectasia (Fig. 10).

**Fig. 11:**
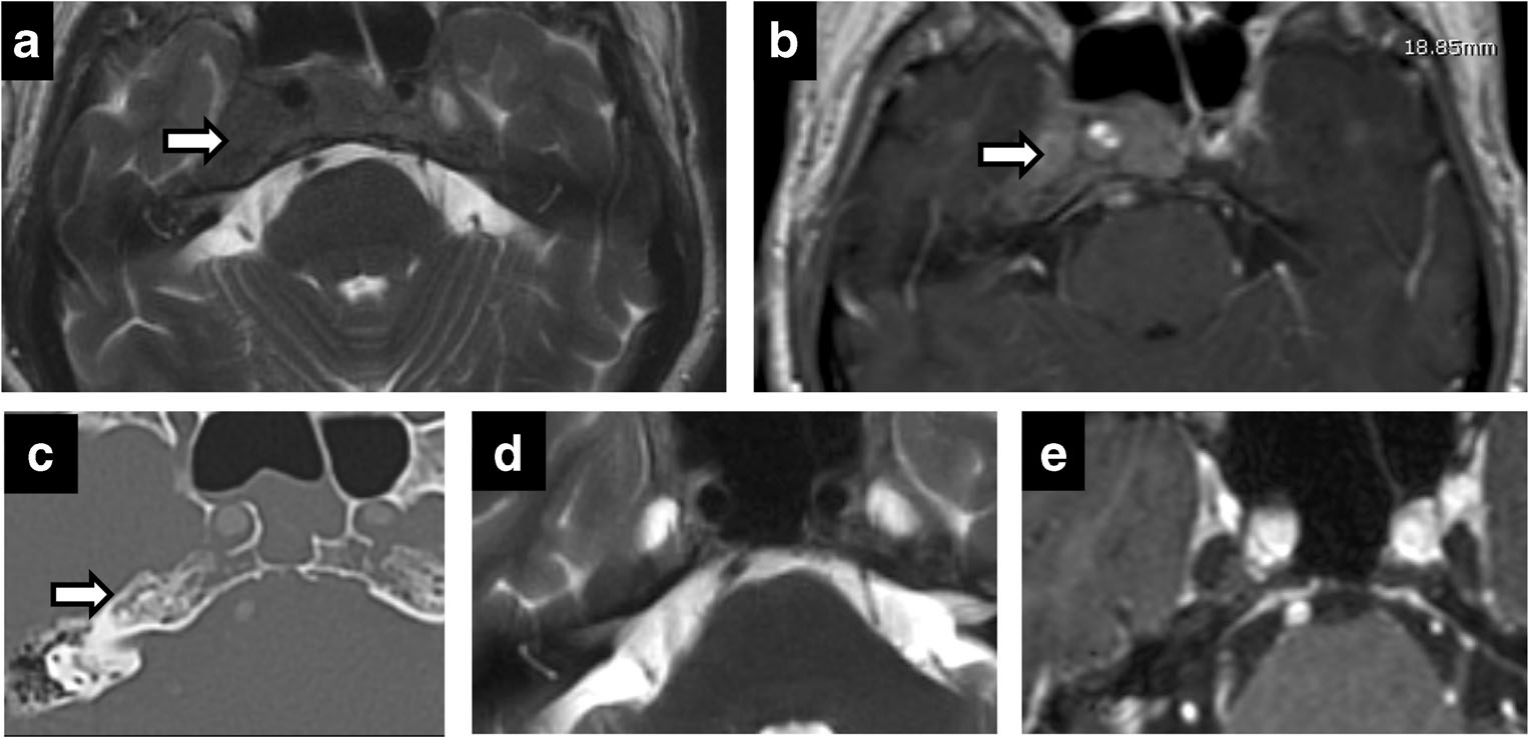
Lymphoma. Axial T2 (**a**), axial C+ (**b**), CT (**c**). Bilateral CN VI palsy and right facial pain. Mottled appearance of clivus and petrous apices on CT—caused by T2 hypointense, enhancing infiltrating lesion. Post treatment (**d**, **e**)—complete resolution

Leptomeningeal metastases, most frequently from breast and lung malignancies, result in linear segmental enhancement, usually in the setting of disseminated disease [22]. Lymphoma can result in neural involvement from either perineural invasion or leptomeningeal disease [2]. Lymphoma can cause dural tail, but absence of hyperostosis helps differentiate it from meningioma (Fig.11). Sequences that highlight CSF such as T2 SPACE or CISS play an important part in helping detect CSF disseminated malignancies. The normal high T2 signal of CSF maybe replaced by low signal from malignant lesions.

**Fig. 12:**
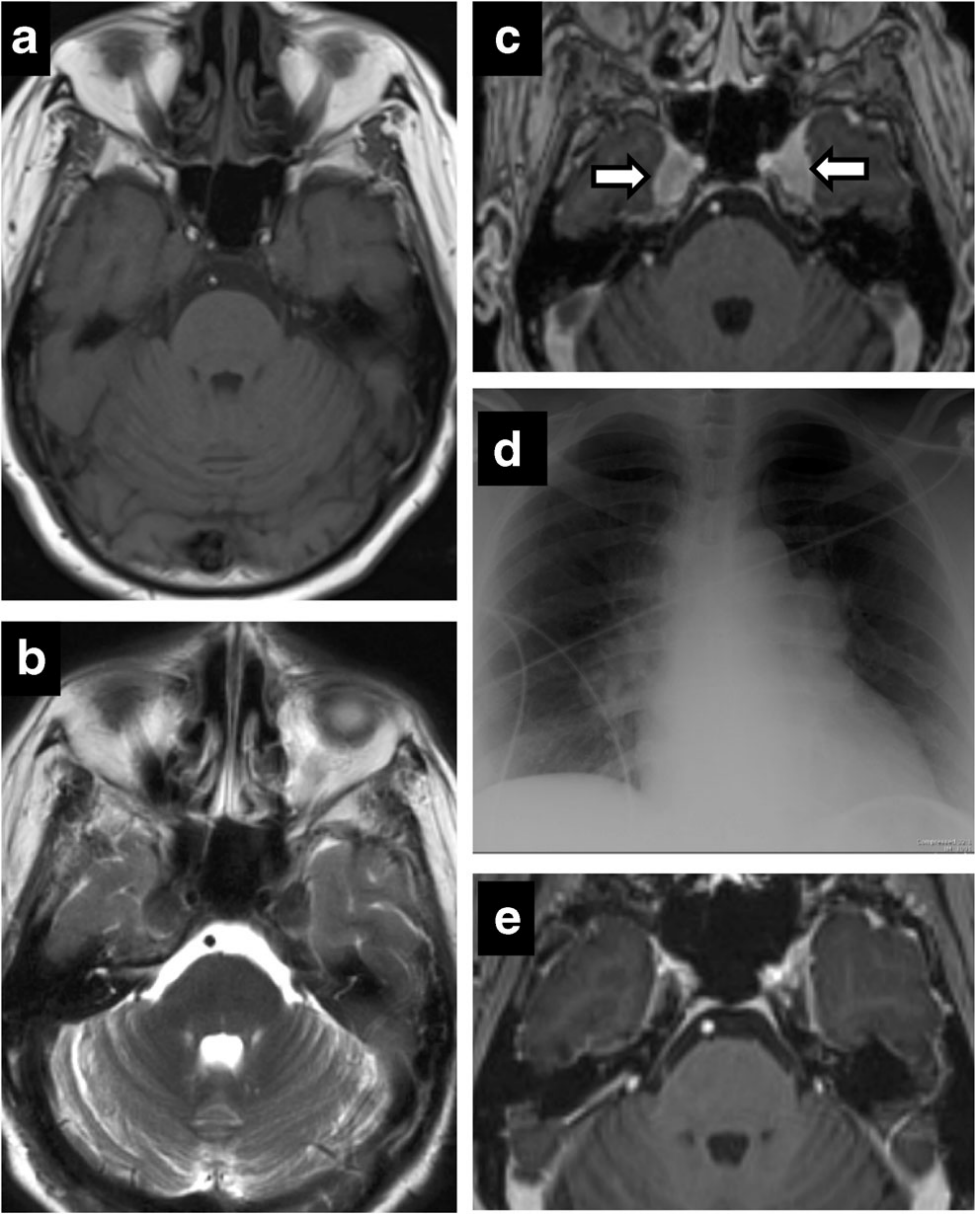
Sarcoidosis. Axial T1 (**a**), axial T2 (**b**), axial T1 C+ (**c**). A 53-year-old woman with facial pain and numbness. Enhancing, T2 hypointense lesions symmetrically involving the bilateral Meckel’s cave. Chest X-ray (**d**)—bilateral hilar and mediastinal lymphadenopathy. Follow-up (**e**)—complete resolution

Inflammatory aetiologies such as sarcoidosis have more nodular enhancement than other leptomeningeal diseases due to granulomas and can involve the trigeminal nerve. Symmetrical involvement of Meckel’s cave is rarely reported [13] (Fig. 12). Neurosarcoidosis is rare without pulmonary manifestations and facial nerve involvement is more common [6, 19]. Additionally, involvement of pituitary hypothalamic axis can help point towards the correct diagnosis.

**Fig. 13:**
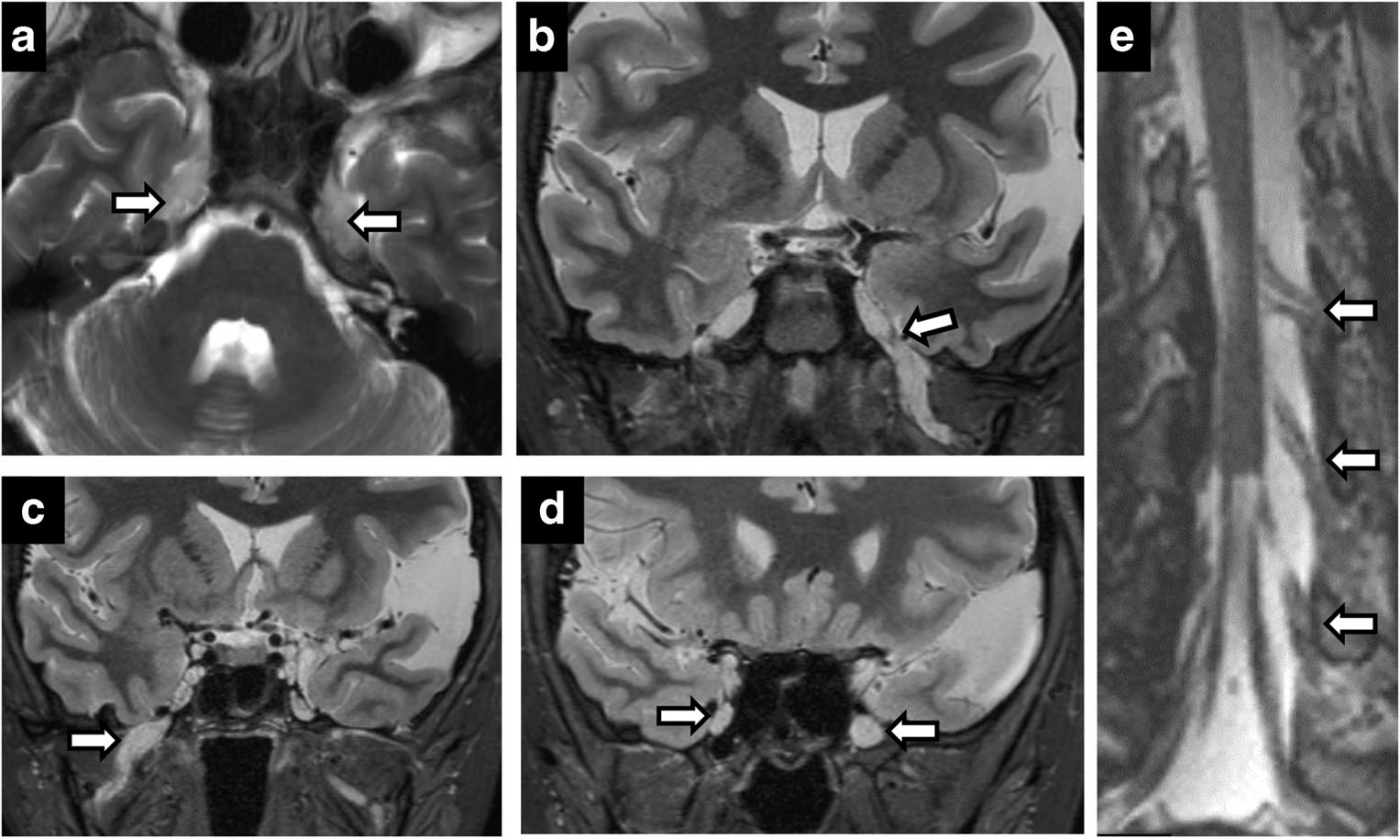
Charcot Marie Tooth (CMT) disease. Fusiform thickened bilateral CN V (**a**-**d**)—including V3 segment extending through Foramen ovale (*arrows* in **b**, **c**) and V2 segment in the foramen rotundum (*arrow* in **d**). Diffusely thickened spinal nerves (**e**)

Diffuse cranial nerve marked enlargement can be seen in chronic inflammatory demyelinating polyneuropathy (CIDP), neurofibromatosis and hereditary sensory motor neuropathies (HSMNs). HSMN type I (Charcot-Marie-Tooth disease) demonstrates no significant enhancement nor leptomeningeal disease [3] (Fig. 13). Diagnosis is frequently known from genetic testing of the autosomal-dominant characteristic clinical history of distal weakness and absent reflexes beginning in the second decade. CIDP demonstrates diffuse enhancement and neurofibromatosis demonstrates more defined mass lesions and numerous additional findings such as plexiform fibromas and sphenoid wing dysplasia.

### Non-trigeminal disease

**Fig. 14:**
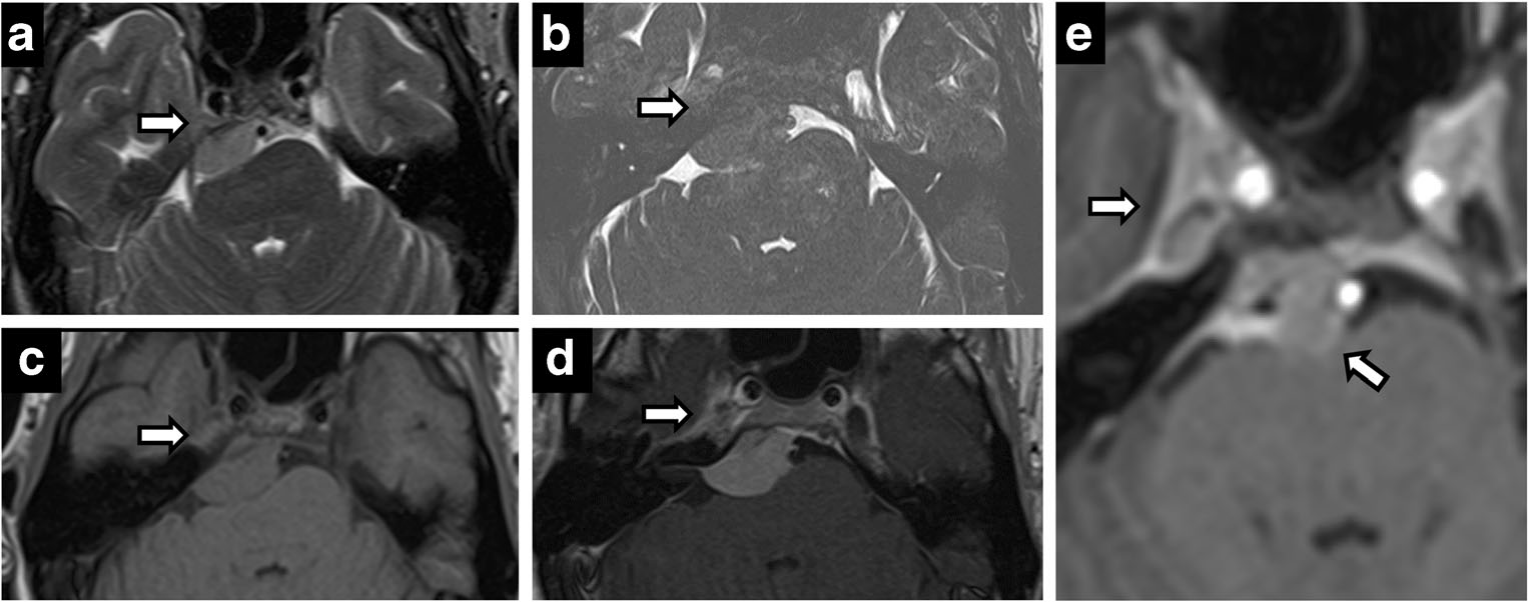
Meningioma (extrinsic). Axial T2 (**a**), T2 SPACE (**b**), axial T1 (**c**), axial C+ (**d**). Right petroclival T2 hypointense, enhancing mass invading the right Meckel’s cave. Postoperative residual enhancing lesion in the right Meckel’s cave and abutting basilar artery (**e**)

Meckel’s cave, being composed of dura, is subject to meningiomas. Meningiomas may originate in the dura mater in or around Meckel’s cave (Fig. 14). Rarely, the tumours can be confined within the cave and arise from the trigeminal nerve [8]. Additionally, the cave may also be invaded by non-neural processes such as posterior extension of pituitary macroadenoma and orbital inflammatory disease. Lesions of adjacent bone and other structures may extrinsically compress the canal, best assessed on thin coronal T2-weighted imaging. Examples include petrous apex, petroclival fissure and clival diseases, osseous expansion from ocular nerve sheath tumours and internal carotid artery (ICA) aneurysms.

**Fig. 15:**
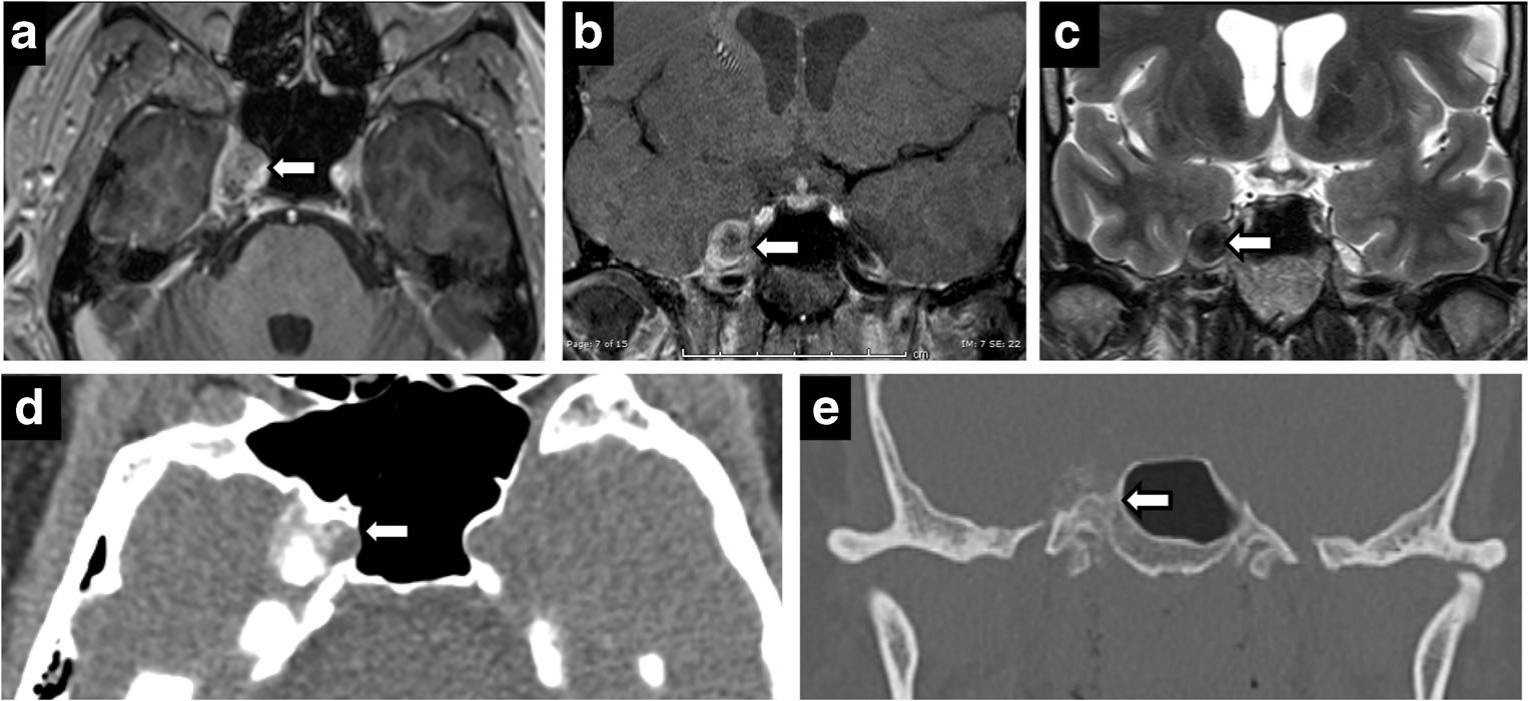
Meningioma (intrinsic). Axial C+ (**a**), coronal C+ (**b**), coronal T2 (**c**), axial CT (**d**), coronal CT (**e**). Circumscribed enhancing right Meckel’s cave mass—hypointense on T2. CT shows calcifications with hyperostosis of adjacent bone

Thin, high-resolution, three-dimensional constructive interference in steady state imaging can distinguish the second most common primary neoplasm in Meckel’s cave, meningioma, from nerve sheath tumour. Meningiomas are peripheral with enhancing dural tail, arising from the dural reflections comprising the cave’s margins, while nerve sheath tumours will be more central within the cave, growing along the course of the nerve. Calcifications and T2-hypointensity in meningiomas are additional distinguishing findings (Fig. 15).

Pituitary macroadenomas can be invasive, contiguously extending from the sella, through the cavernous sinus, to Meckel’s cave. Such large tumours are associated with sellar expansion, ICA encasement without extrinsic compression, sphenoid sinus extension, and are relatively homogeneous and moderately T2-hyperintense.

**Fig. 16:**
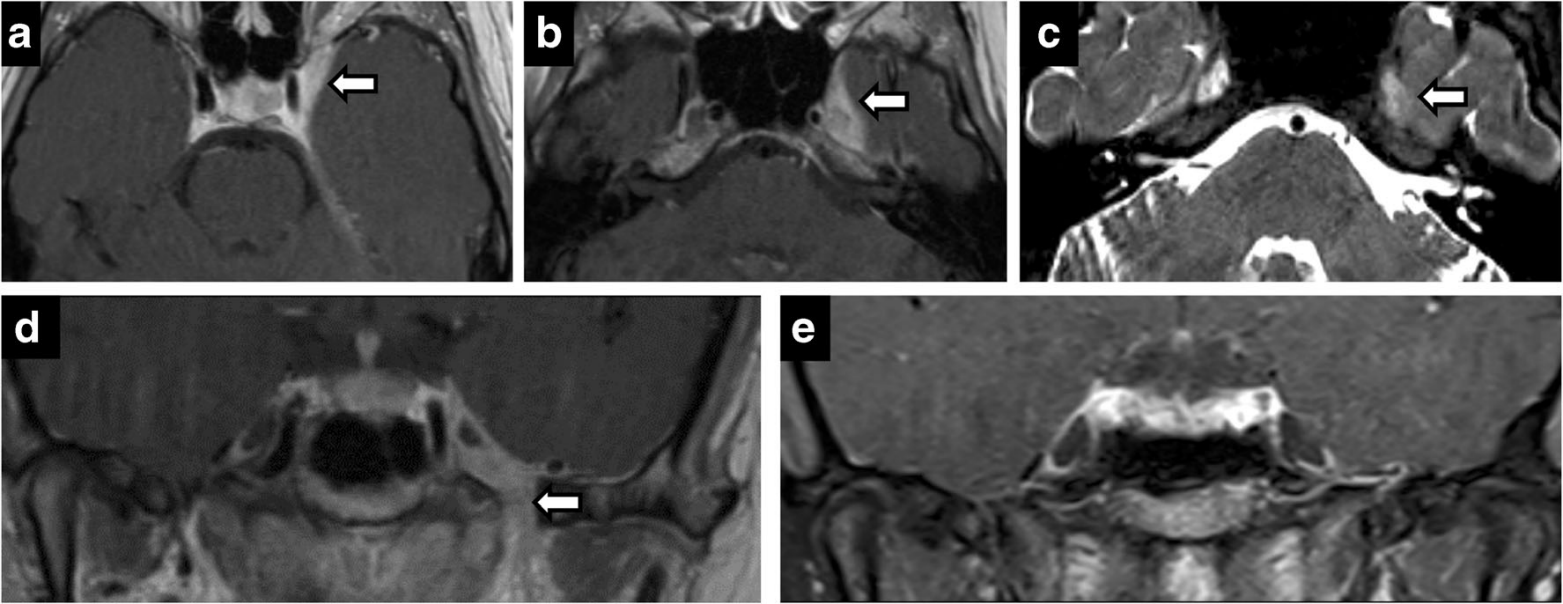
Tolosa-Hunt syndrome. Axial T1 C+ (**a**, **b**), axial T2 (**c**), coronal C+ (**d**). A 23-year-old woman with acute onset of painful left diplopia. Asymmetric enhancing tissue in left cavernous sinus extending to Meckel’s cave and through the foramen ovale (**b**, **d**). Follow-up—complete resolution (**e**)

Posterior extension of pathology to Meckel’s cave can also be seen in Tolosa Hunt, a variant of orbital inflammatory disease (orbital pseudotumour) involving the orbital apex that extends posteriorly into the cavernous sinus [7]. Patients present with painful ophthalmoplegia and cavernous fullness, asymmetric enhancement and ICA narrowing (Fig. 16).

**Fig. 17:**
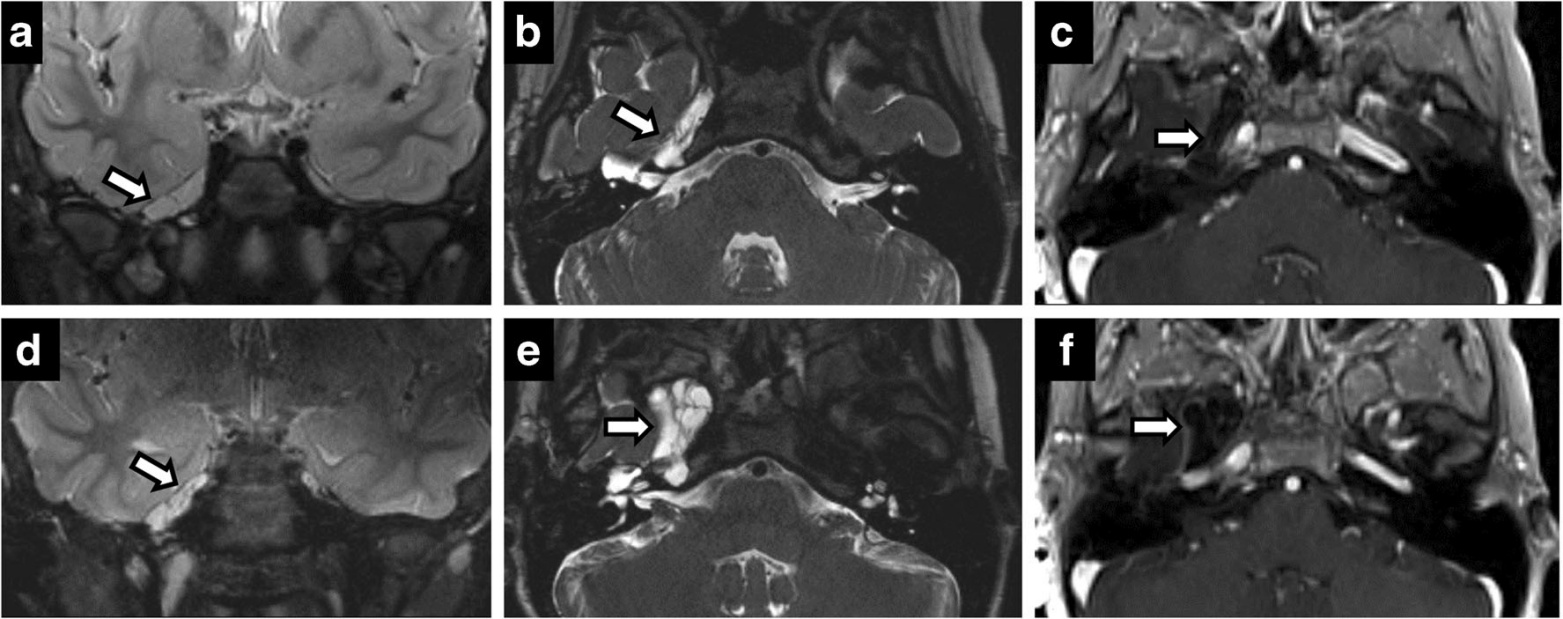
Petrous apex cephalocele. Coronal (**a**, **d**) and axial (**b**, **e**) T2-weighted images of a cystic petrous apex lesion that communicates with the posterolateral portion of Meckel’s cave. No abnormal enhancement on axial post-contrast images (**c**, **f**)

**Fig. 18:**
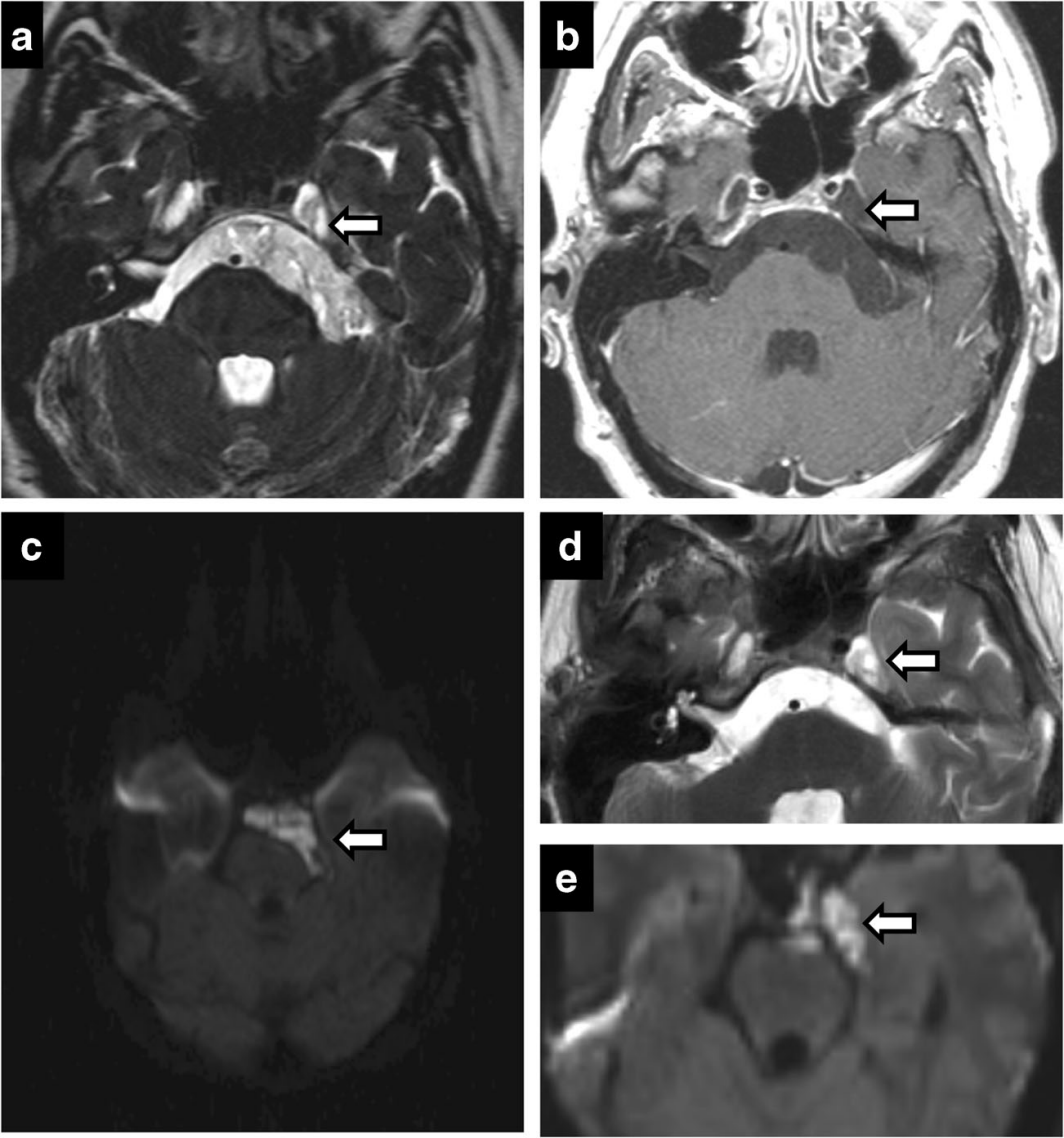
Epidermoid. Expansion of left Meckel’s cave by a left cerebellopontine angle mass on axial T2 (**a**), showing no enhancement (**b**) and restricted diffusion (**c**). Postoperative residual tissue in left Meckel’s cave (**d**, **e**)

**Fig. 19:**
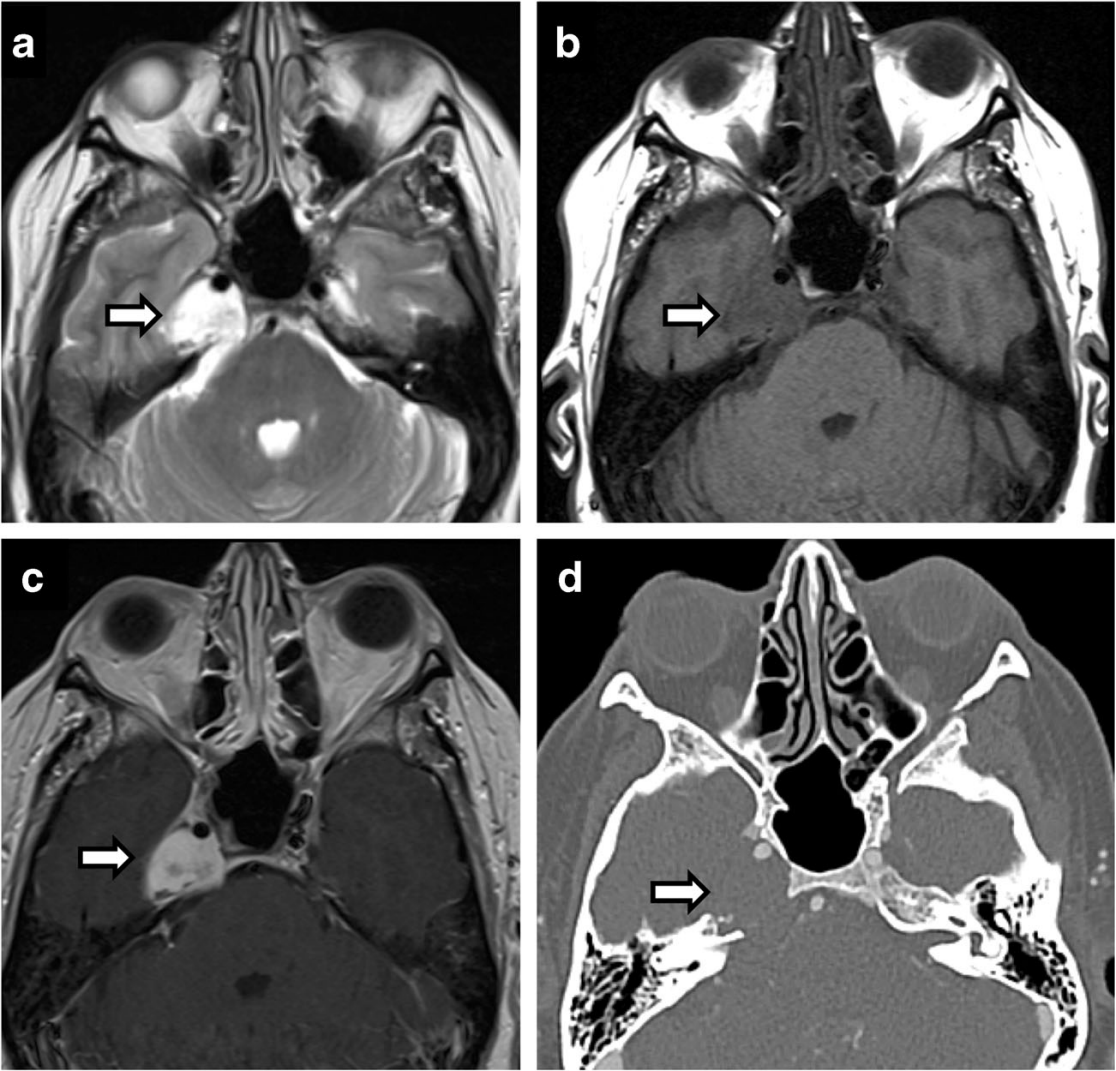
Chondrosarcoma. Axial T2 (**a**), axial T1 (**b**), axial T1 C+ (**c**), CT (**d**). A 61-year-old woman with effacement of the right Meckel’s cave by an expansile petrous apex mass that is hyperintense on T2, hypointense on T1 and shows avid enhancement on post contrast image. CT shows features of slow growing lesion. Note preserved Meckel’s cave on the left

Osseous processes compressing Meckel’s cave involve the petrous apex, petroclival fissure and clivus. Petrous apex cephalocele is usually an incidental finding, reflecting benign ballooning of the arachnoid space communication with Meckel’s cave. Fluid distended Meckel’s cave has an enlarged porus trigeminus notch and a smooth expansile cystic space in the anteromedial petrous apex [20] (Fig. 17). Findings may reflect intracranial hypertension, similar to empty sella, and is associated with spontaneous CSF leaks due to dehiscence [1]. Signal characteristics follow CSF, with FLAIR suppression. Petrous apex mucoceles have a similar appearance but do not connect to Meckel’s cave; rather, they compress it, resulting in symptoms. Cholesterol granulomas are T1 hyperintense, demonstrate susceptibility and no FLAIR suppression. Congenital cholesteatomas and epidermoids both demonstrate restricted diffusion, incomplete FLAIR suppression and no enhancement, but congenital cholesteatomas are localised to the petrous apex, while epidermoids are in the cerebellopontine angle/prepontine cistern and are much more proliferative, extending into multiple cisterns and encasing the basilar artery [14] (Fig. 18). Petroclival/petrooccipital fissure chondroid lesions demonstrate characteristic imaging features on CT with rings and arcs calcifications (Fig. 19). Clival chordomas demonstrate extensive bony destruction, marked T2-hyperintensity, haemorrhagic and calcific susceptibility, and honeycomb enhancement pattern. They can be distinguished from pituitary macroadenoma by lack of sellar mass, sparing of the sphenoid sinus and signal characteristics. There are isolated case reports of intradural chordomas of the Meckel’s cave and paraganglioglioma.

**Fig. 20:**
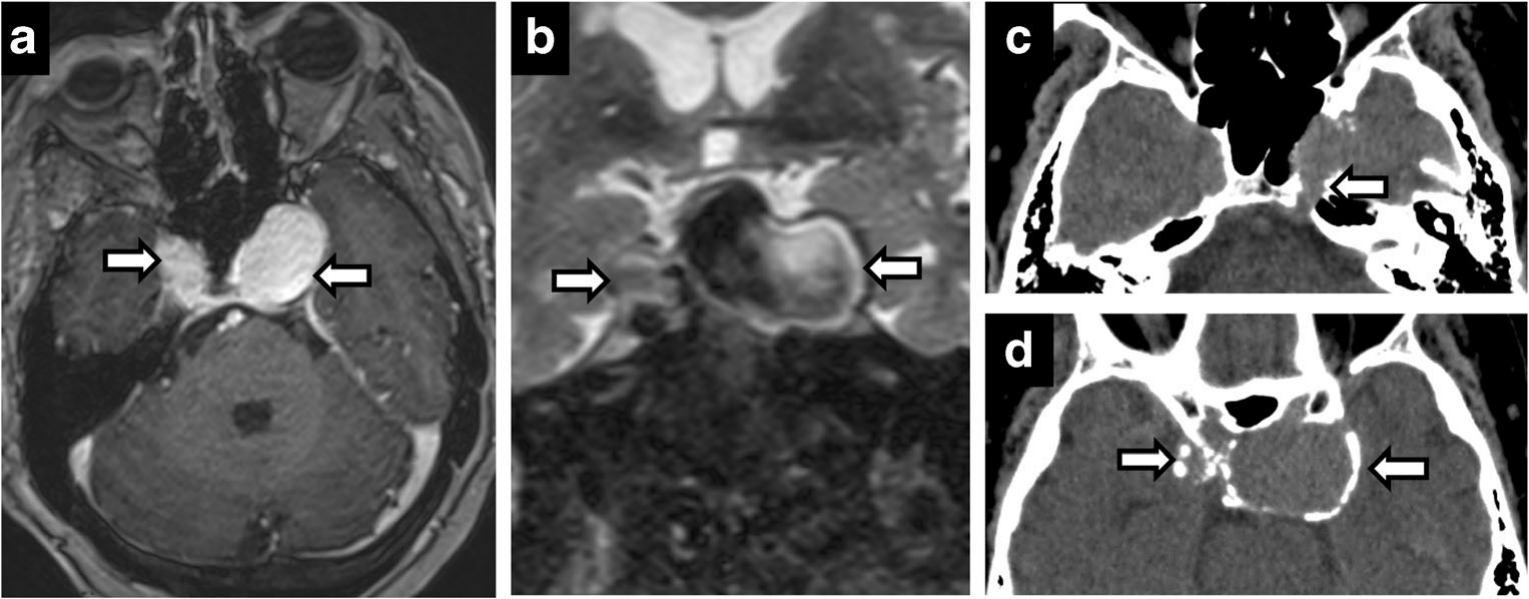
Bilateral cavernous ICA aneurysms. Axial C+ (**a**), coronal T2 (**b**), CT (**c**) and (**d**). A 73-year-old woman with left facial pain. Bilateral cavernous enhancing, partially calcified lesions encroaching the Meckel’s cave—left greater than right

Aneurysms of the petrous and cavernous segments of the ICA can result in mass effect on Meckel’s cave given the close proximity (Fig. 20). These segments are demarcated by the petrolingual ligament. Ruptured aneurysms in either location do not cause subarachnoid haemorrhage as they are extradural, but cavernous ruptured aneurysms and dissections may cause carotid-cavernous fistulas.

## Conclusions

Meckel’s cave contains the trigeminal nerve ganglion and proximal rootlets, situated between the prepontine cistern and the cavernous sinus. Key imaging features of pathology of Meckel’s cave are effacement of CSF signal in Meckel’s cave, moderate enhancement greater than the perineural vascular plexus, nerve enlargement with perineural fat plane effacement and osseous foraminal erosion or enlargement. Neural pathologies include nerve sheath tumours, perineural tumour spread, viral/idiopathic neuralgia, leptomeningeal metastases, sarcoidosis and polyneuropathies such as CIDP and HMSN. Non-neural pathologies include: pituitary macroadenoma; Tolosa Hunt; petrous, petroclival and clival lesions; vessel mass effect.
